# Mapping COVID vulnerability in relation to drug and alcohol recovery residence availability across the United States

**DOI:** 10.1186/s12889-023-17048-x

**Published:** 2023-11-17

**Authors:** Amy A. Mericle, Deidre Patterson, Meenakshi S. Subbaraman, Jason Howell, Dave Sheridan, Thomasina Borkman, Jayla Burton, Katherine J. Karriker-Jaffe

**Affiliations:** 1 Alcohol Research Group at the Public Health Institute, 6001 Shellmound St., Suite 450, Emeryville, CA 94608 USA; 2 Behavioral Health and Recovery Studies at the Public Health Institute, Oakland, CA USA; 3 Recovery People, Elgin, TX USA; 4 National Alliance for Recovery Residences, St. Paul, MN USA; 5 George Mason University, Fairfax, VA USA; 6 Community Health & Implementation Research Program at RTI International, Berkeley, CA USA

**Keywords:** Recovery housing, Recovery residences, COVID, COVID vulnerability, Social determinants of health

## Abstract

Recovery housing is an important resource for those in recovery from substance use disorders. Unfortunately, we know little about its relationship to key community health risk and protective factors, potentially limiting the role it could play as a broader health resource. Leveraging county-level data on recovery residences from the National Study of Treatment and Addiction Recovery Residences (NSTARR), this study used multilevel modeling to examine Community COVID Vulnerability Index (CCVI) scores as well as availability of COVID testing and vaccination sites in relation to recovery housing. CCVI composite scores were positively associated with recovery housing availability. Analyses using CCVI thematic sub-scores found that population density and number of churches were positively associated with recovery housing availability, while epidemiological factors and healthcare system factors were negatively associated with recovery housing availability. In counties with recovery housing, there also was a positive association between CCVI and both COVID testing and vaccination availability. Recovery residences tend to be located in areas of high COVID vulnerability, reflecting effective targeting in areas with higher population density, more housing risk factors, and other high-risk environments and signaling a key point of contact to address broader health issues among those in recovery from substance use disorders.

## Introduction

Early in the coronavirus disease (COVID) pandemic, persons with substance use disorders (SUD) were identified to be at increased risk for SARS-CoV-2 infection [1, 2]. However, many of the risks to persons with SUD are indirect and arise from factors such as housing instability and incarceration, as well as reduced access to healthcare and other services [3]. These factors are examples of social determinants of health (SDOH), which affect a wide range of health, functioning, and quality-of-life outcomes through direct and indirect pathways [4]. The COVID pandemic has magnified socioeconomic disparities in health, sparked renewed calls to incorporate SDOH into epidemiological modeling of infectious diseases, and underscored the need for more effective policies and programs to address these environmental conditions that drive many health outcomes [5, 6], including recovery from drug and alcohol problems [7].

In addition to risk for COVID infection, the pandemic also may have increased risk for return to use among those in recovery from SUD, as general population studies have highlighted increased levels of alcohol [8] and substance use [9] as well as increased overdose risk [10–12] and use of alcohol and other substances as a means to cope during the pandemic [13, 14]. Although data from a national survey of adults with resolved alcohol use disorder (*N* = 1,492) found that equivalently large majorities of women and men reported that the COVID pandemic had not affected their recovery at all (88.9% and 88.8%, respectively), a shorter length of time in recovery was associated with increased risk of return to use during the pandemic among women [15]. Changes in substance use patterns, SUD and overdose risk during the pandemic were not equally distributed, with evidence of stark disparities by gender, race and ethnicity [16, 17].

Safe and supportive housing is critical to recovery from SUD [18]. Recovery housing is a community-based intervention that has been found to increase recovery capital [19] and addresses a critical SDOH, stable housing. Recovery residences go by a variety of names (e.g., Oxford Houses™, sober living houses, recovery homes, halfway houses, therapeutic communities) and can range from those based solely on mutual aid to those that also provide clinical services. Reviews of evidence on recovery housing consistently highlight positive outcomes [20, 21], and findings to define recovery housing evidence-based practices (EBPs) are beginning to emerge [22, 23].

By providing housing and other support, recovery residences are a key component of comprehensive, recovery-oriented systems of care [24–26]; they may also contribute to more recovery-ready communities [27] and build our understanding of the ecology of recovery [28] from SUD. Unfortunately, little is known about recovery housing in relation to health risk and healthcare resources–two factors highly relevant in the COVID pandemic. Although one study found a lower percentage of COVID infection and mortality in Oxford House residents than in the general population [29], studies of the availability of recovery housing at the local [30], state, and national levels [31] show recovery residences are not distributed evenly, which may have important implications for understanding and potentially mitigating COVID risk and other downstream, pandemic-related effects among individuals living in recovery residences.

To better understand these risks, this study examines community-level COVID vulnerability in relation to US recovery residences. Specifically, we explore whether COVID vulnerability is associated with recovery housing availability and density at the county-level as well as which factors driving COVID vulnerability are most strongly related to availability and density. To better understand the availability of health resources in the communities where recovery residences are located, we also present data on availability and accessibility of COVID testing and vaccination services and explore how this is related to overall COVID vulnerability and the factors that comprise it.

## Methods

Using geolocated data on recovery residences from the National Study of Treatment and Addiction Recovery Residences (NSTARR) database and the Community COVID Vulnerability Index (CCVI), this secondary data analysis study used multilevel modeling to examine whether the CCVI and other community resources were related to recovery housing availability and density. In counties with at least one recovery residence, we also used multilevel modeling to examine whether the CCVI and other community resources were associated with density of and mean distances of recovery housing from COVID testing and vaccination sites at the county level using US Census Bureau’s 2019 County Business Patterns data. The NSTARR COVID Supplement study was reviewed, approved, and monitored by the Public Health Institute Institutional Review Board, which determined it to be exempt from review under Category 2 of 45 CFR 46.104. Details pertaining to our data sources and analytic approach are provided below.

### Description of the NSTARR database

Recovery housing data came from the NSTARR project, which collected information on locations of recovery residences during 2020. We used an NSTARR database export from spring 2021 containing information on 10,358 residences operated by 3,628 providers in all 50 states [31]. *Recovery housing availability* was operationalized as an indicator of any recovery housing (vs. none) in a given county and *recovery housing density* was a count of recovery residences in each county that had any recovery housing.

### Description of COVID-related contextual variables

#### 2020 Community COVID Vulnerability Index (CCVI)

The 2020 CCVI is a composite measure of seven social determinants of health, encompassing modified themes from the Centers for Disease Control and Prevention’s Social Vulnerability Index in combination with COVID risk factors to identify communities in need of additional support during the COVID pandemic. There is an overall score, as well as thematic scores for each domain (Socioeconomic status; Minority status and language; Housing type, transportation, household composition and disability; Epidemiological factors; Healthcare system factors; High-risk environments; Population density). Each county was ranked on a scale from 0 = least vulnerable to 1 = most vulnerable on each of 40 variables, which then were summed by theme, with scores ranging from 0–1 [32, 33].

#### COVID testing and vaccination sites

As has been used in other studies examining access to COVID testing and vaccination sites [34–36] we utilized point data on COVID testing and vaccination site locations from the Urban and Regional Information Systems Association (URISA) GISCorps “COVID Vaccination and Testing Provider Locations in the United States” dataset**.** The crowd-sourced data were collected between March 2020 and November 2021. All data collection, cleaning, and validation tasks were completed by a group of over 300 GISCorps volunteers. We removed duplicates and selected non-private testing (*n* = 16,989) and vaccination (*n* = 4,605) sites for our project, as these would be accessible to people regardless of health insurance provider or coverage status. The data on testing and vaccination sites came as a geocoded shapefile.

#### Other community resources

To supplement the testing and vaccination site data, we appended county-level counts of community resources from the US Census Bureau’s 2019 County Business Patterns dataset. We included count data on two specific types of healthcare providers likely to provide testing and vaccination for COVID: physicians’ offices (North American Industry Classification System [NAICS] codes = 62,111, 62,112) as well as pharmacies and drug stores (NAICS codes = 446,110). We also included schools and churches because public testing sites and vaccination efforts often were conducted at elementary and secondary schools (NAICS codes = 611,110), community colleges (NAICS codes = 611,210) and churches (NAICS codes = 813,110) to expand the reach of public health efforts during the pandemic [37, 38].

### Analyses

#### Geocoding process & geographic measures

Locations of all recovery residences were geocoded using ArcGIS [39], with 86.4% of recovery residences successfully matched at either the street or ZIP code level (the remainders were unmatched due to missing address information). The largest portion of geocoded recovery residences (51%) were located in the Southern region of the US due to a sizable number of residences in Florida, with 15% located in the Northeast region, 17% located in the Midwest, and 17% located in the West. County-level Federal Information Processing System (FIPS) codes were used to append county-level data on COVID vulnerability and community resources. The point-level data were used by the authors to map locations of each recovery residence in relation to nearby COVID testing and vaccination sites. Measures derived for analysis were the county-level counts of testing sites and vaccination sites (availability measures), as well as the distance of each recovery residence to the nearest testing site and to the nearest vaccination site (accessibility measures; see Table 1). The distance-to-nearest measures were calculated using Network Analyst Extension from ArcGIS.

#### Bivariate and multivariable analyses

Bivariate t-tests for preliminary analyses compared COVID vulnerability scores, density of testing and vaccination sites, and other community resources in counties with and without recovery housing. Associations between COVID vulnerability and community resources with recovery housing availability and recovery housing density were examined using multilevel logistic and negative binomial regression models. The dichotomous indicator of recovery housing availability (any vs. none, *N* = 3,142 counties) and basic count variable of recovery housing density were specified as outcomes in separate regression models, with the density models limited to counties with at least one recovery residence (*n* = 2,213 counties). The series of multilevel regression models began with unadjusted models using the continuous overall CCVI composite score as the independent variable for both outcomes. Adjusted models then simultaneously tested all seven CCVI theme domain scores and included density of community resources as continuous independent variables. All models included a fixed effect for state and adjustment of the standard errors to account for clustering at the state level. Another set of negative binomial regression models followed the same series, using as the outcome variable the county-level counts of testing and vaccination sites in those counties with at least one recovery residence (*n* = 2,213 counties). These were followed by a final set of linear regression models using mean distances to testing and vaccination sites from recovery housing (also in the subset of counties with at least one recovery residence). All regressions were run in Stata 16.1 [40].

## Results

Compared to counties without recovery housing, counties with recovery housing had significantly higher scores on the composite CCVI total score, as well as significantly higher scores on subdomains covering minority status and language, high-risk environments, and population density, but significantly lower scores on subdomains covering socioeconomic status, epidemiological factors related to COVID transmission, and healthcare system factors (see Table 1). Counties with recovery housing also had markedly higher densities of non-private COVID testing sites, higher densities of non-private COVID vaccination sites, and greater levels of community resources (across all categories examined) than counties without recovery housing.

**Table 1:** US County-level descriptive statistics in counties with and without recovery housing

	**Full sample**	**Counties with Recovery Housing**	**Counties with No Recovery Housing**	
	*N* = *3,142*	*n* = *929*	*n* = *2,213*	*p-value*
**Vulnerability Index (0 to 1)**^**a**^				
COVID Community Vulnerability Index (CCVI)	0.50 (0.29)	0.55 (0.25)	0.48 (0.30)	< 0.001
*CCVI Theme 1*: Socioeconomic status	0.50 (0.29)	0.48 (0.26)	0.51 (0.30)	0.002
*CCVI Theme 2*: Minority status & language	0.50 (0.29)	0.60 (0.27)	0.46 (0.29)	< 0.001
*CCVI Theme 3*: Housing type, transportation, household composition & disability	0.50 (0.29)	0.50 (0.27)	0.50 (0.30)	0.823
*CCVI Theme 4*: Epidemiological factors	0.50 (0.29)	0.36 (0.27)	0.56 (0.27)	< 0.001
*CCVI Theme 5*: Healthcare system factors	0.50 (0.29)	0.47 (0.28)	0.51 (0.29)	< 0.001
*CCVI Theme 6*: High-risk environments	0.50 (0.29)	0.57 (0.27)	0.47 (0.29)	< 0.001
*CCVI Theme 7*: Population density	0.50 (0.29)	0.75 (0.21)	0.39 (0.25)	< 0.001
**Testing and Vaccination Sites**				
Number of non-private COVID testing sites	5.38 (15.72)	12.91 (27.13)	2.22 (2.88)	< 0.001
Distance of recovery housing to nearest non-private COVID testing site in miles		3.66 (5.74)	–-	
Number of non-private COVID vaccine sites	1.46 (4.04)	3.41 (6.81)	0.65 (1.22)	< 0.001
Distance of recovery housing to nearest non-private COVID vaccine site in miles		16.73 (26.64)	–-	
**Community Resources**				
Number of physicians’ offices	68.24 (294.78)	197.82 (512.36)	12.41 (30.87)	< 0.001
Number of pharmacies & drug stores	14.14 (50.97)	38.49 (87.77)	3.64 (5.93)	< 0.001
Number of elementary & secondary schools	6.79 (28.59)	20.19 (49.40)	1.02 (2.85)	< 0.001
Number of community colleges	0.15 (1.15)	0.51 (2.04)	0.00 (0.00)	< 0.001
Number of churches	59.94 (126.78)	142.24 (205.39)	24.47 (25.63)	< 0.001

All values are mean (standard deviation). *P*-values are for unadjusted tests of mean differences

^a^Values for the CCVI total and for each CCVI theme sub-score were standardized nationally, so there is no variation in the national county-level scores

### COVID vulnerability

**Fig. 1:**
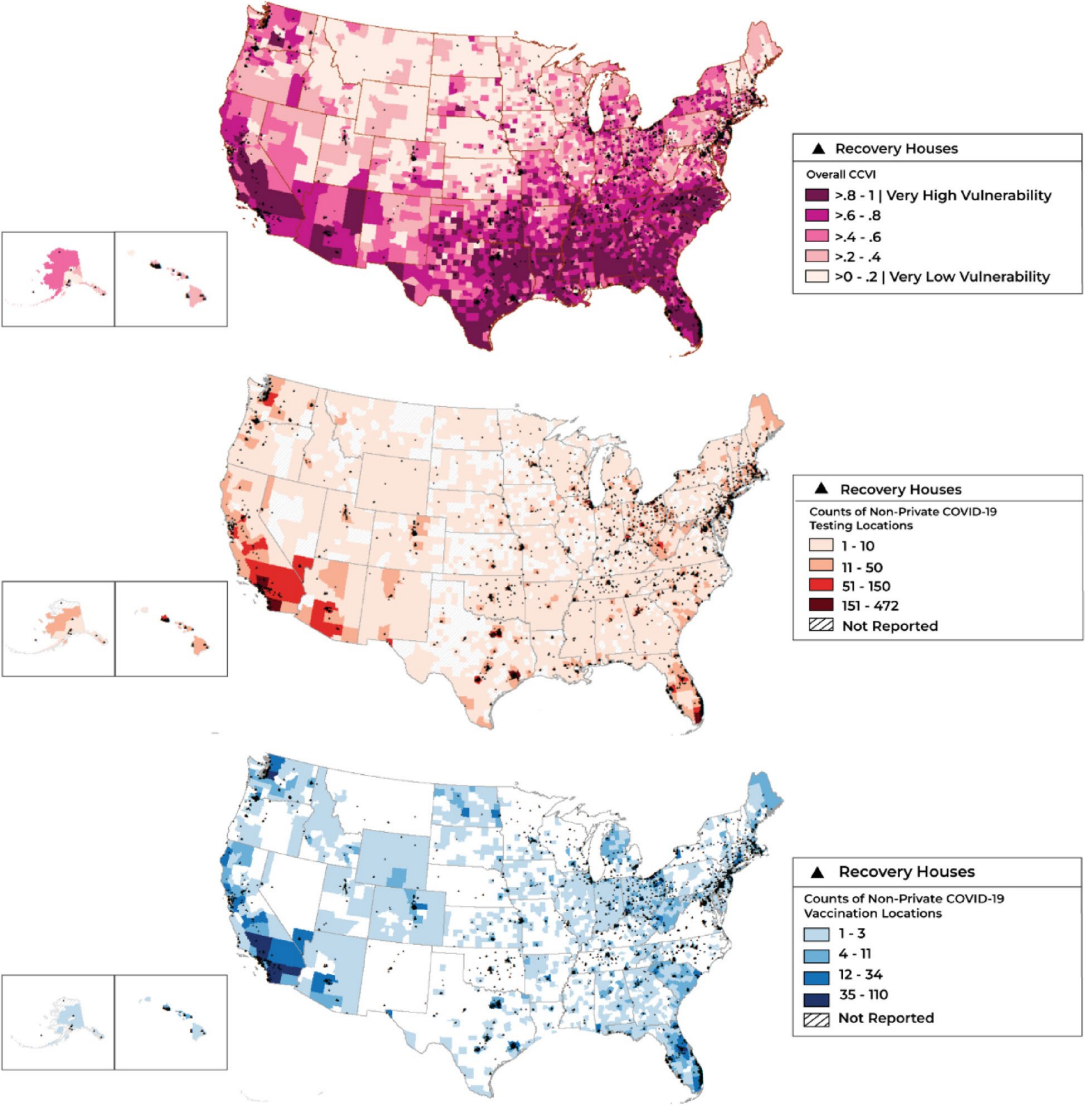
County-level COVID vulnerability, testing and vaccination resources, and recovery housing across the US. *Caption*. Maps were created by the authors within ArcGIS Desktop (Release 10.8.1; https://www.esri.com/en-us/arcgis/products/arcgis-desktop/overview). Dots on all maps depict recovery housing. The top left map displays recovery housing in relation to levels of COVID vulnerability measured by the overall CCVI score (darker areas have greater COVID vulnerability). The top right map displays recovery housing in relation to density of non-private COVID testing, and the bottom map displays recovery housing in relation to density of non-private COVID vaccination locations (darker areas have greater density of COVID resources on both maps)

Figure 1 depicts county-level COVID vulnerability in relation to recovery housing across the US. In the regression models (Table 2), the CCVI composite scores were positively associated with recovery housing availability (whether the county had any recovery housing versus none; OR [95% CI] = 17.02 [10.01, 28.94]; *p* < 0.001) and recovery housing density in counties with at least one recovery residence (prevalence rate ratio, PRR [95% CI] = 6.23 [3.78, 10.28]; *p* < 0.001). When the separate thematic sub-scores were entered simultaneously along with availability of community resources, population density and the number of churches were positively associated with both measures of recovery housing availability, while epidemiological factors and healthcare system factors were negatively associated with both measures of recovery housing availability. Housing risk factors and high-risk environments were positively associated with presence of any recovery housing. The minority status and language score was positively associated with recovery housing density, and the number of drug stores and pharmacies was negatively associated with recovery housing density. No associations were observed between recovery housing with the socioeconomic status score, the number of physicians’ offices, or the number of educational institutions (neither elementary and secondary schools nor community colleges) in the county.

**Table 2:** Logistic and negative binomial regression results for COVID Community Vulnerability Index (CCVI) regressed on county-level recovery housing availability (any vs. none; *N* = 3,142 counties) and density of recovery housing in counties with at least one recovery residence (*n* = 2,213 counties)

	Any Recovery Housing		Count of Recovery Houses	
	Odds Ratio (95% CI)		Prevalence Rate Ratio (95% CI)	
**Unadjusted**				
CCVI Composite Score	17.020***	(10.010, 28.937)	6.231***	(3.777, 10.280)
**Adjusted**				
*CCVI Theme 1*: Socioeconomic Status	1.358	(0.563, 3.277)	1.529	(0.921, 2.539)
*CCVI Theme 2*: Minority Status & Language	1.217	(0.611, 2.425)	2.427***	(1.562, 3.770)
*CCVI Theme 3*: Housing Type & Household Composition	3.501***	(1.763, 6.952)	0.966	(0.650, 1.436)
*CCVI Theme 4*: Epidemiological Factors	0.115***	(0.050, 0.268)	0.626*	(0.411, 0.953)
*CCVI Theme 5*: Healthcare System Factors	0.302*	(0.118, 0.771)	0.239***	(0.156, 0.367)
*CCVI Theme 6*: High Risk Environments	2.015*	(1.095, 3.708)	0.821	(0.583, 1.158)
*CCVI Theme 7*: Population Density	61.360***	(23.869, 157.739)	9.776***	(6.399, 14.936)
Physicians’ Offices	1.000	(0.993, 1.007)	1.000	(0.999, 1.001)
Drug Stores & Pharmacies	1.031	(0.989, 1.074)	0.998*	(0.996, 1.000)
Elementary & Secondary Schools	1.020	(0.978, 1.063)	0.999	(0.994, 1.005)
Community Colleges	–-^a^		1.004	(0.945, 1.067)
Churches	1.013**	(1.004, 1.022)	1.003***	(1.002, 1.004)

Separate regression models used for unadjusted and adjusted associations. All models include fixed effects for state and clustered standard errors. Regressions were run in Stata 16.1 using melogit and menbreg commands. ^a^ None of the counties without recovery housing had a community college, so this variable was dropped from this model

*CCVI* COVID Community Vulnerability Index

*CI* Confidence Interval

^*^p < .05 **p < .01 ***p < .001

### Proximity/distance to testing and vaccination sites

Figure 1 also depicts county-level density of non-private COVID testing locations as well as county-level density of non-private COVID vaccination locations, each in relation to recovery housing across the US. In counties with at least one recovery residence, regression models (Table 3) showed a positive association between the CCVI composite score and both testing and vaccination availability (PRR [95% CI] = 8.20 [5.40, 12.45] and 6.18 [3.83, 9.99], respectively; both *p* < 0.001). The composite scores also were negatively associated with average distance to testing and vaccination sites (β [95% CI] = -4.00 [-6.08, -1.93], *p* < 0.001; and -9.89 [-17.92, -1.87], *p* < 0.05, respectively), with shorter mean distances indicative of greater accessibility of COVID testing and vaccination.

**Table 3:** Negative binomial and linear regression results for COVID Community Vulnerability Index (CCVI) regressed on county-level counts of and mean distance to testing and vaccination sites from recovery housing in counties with at least one recovery residence (*n* = 2,213 counties)

	Count of Non-private Testing Locations		Count of Non-private Vaccination Sites	
	Prevalence Rate Ratio (95% CI)		Prevalence Rate Ratio (95% CI)	
**Unadjusted**				
CCVI Composite Score	8.200***	(5.400, 12.451)	6.184***	(3.829, 9.986)
**Adjusted**				
*CCVI Theme 1*: Socioeconomic Status	1.992***	(1.378, 2.880)	2.474**	(1.366, 4.478)
*CCVI Theme 2*: Minority Status & Language	2.171***	(1.563, 3.015)	1.888**	(1.166, 3.059)
*CCVI Theme 3*: Housing Type & Household Composition	1.265	(0.927, 1.725)	0.894	(0.547, 1.461)
*CCVI Theme 4*: Epidemiological Factors	0.631**	(0.460, 0.864)	0.937	(0.564, 1.557)
*CCVI Theme 5*: Healthcare System Factors	0.574**	(0.412, 0.801)	0.321***	(0.191, 0.540)
*CCVI Theme 6*: High Risk Environments	0.584***	(0.467, 0.731)	0.530***	(0.365, 0.770)
*CCVI Theme 7*: Population Density	6.877***	(4.713, 10.036)	8.640***	(4.834, 15.444)
Physicians’ Offices	1.000	(0.999, 1.000)	0.999*	(0.999, 1.000)
Drug Stores & Pharmacies	1.000	(0.998, 1.001)	1.001	(0.999, 1.003)
Elementary & Secondary Schools	1.000	(0.996, 1.004)	0.997	(0.993, 1.001)
Community Colleges	1.018	(0.986, 1.051)	1.025	(0.971, 1.083)
Churches	1.003***	(1.002, 1.004)	1.003***	(1.002, 1.004)
	Mean Distance to Nearest Non-private Testing Location		Mean Distance to Nearest Non-private Vaccination Site	
	Beta (95% CI)		Beta (95% CI)	
**Unadjusted**				
CCVI Composite Score	-4.002***	(-6.078, -1.927)	-9.891*	(-17.916, -1.865)
**Adjusted**				
*CCVI Theme 1*: Socioeconomic Status	-0.375	(-3.341, 2.590)	2.366	(-9.067, 13.798)
*CCVI Theme 2*: Minority Status & Language	-1.859	(-4.325, 0.607)	-5.552	(-13.632, 2.528)
*CCVI Theme 3*: Housing Type & Household Composition	-2.525*	(-4.996, -0.054)	-2.976	(-12.116, 6.163)
*CCVI Theme 4*: Epidemiological Factors	1.808	(-0.684, 4.300)	-8.928	(-20.267, 2.411)
*CCVI Theme 5*: Healthcare System Factors	3.060**	(0.802, 5.319)	-11.346*	(-21.618, -1.073)
*CCVI Theme 6*: High Risk Environments	0.281	(-1.306, 1.869)	4.294	(-2.436, 11.024)
*CCVI Theme 7*: Population Density	-10.054***	(-13.684, -6.423)	-48.520***	(-63.610, -33.430)
Physicians’ Offices	0.000	(-0.001, 0.001)	-0.003	(-0.008, 0.002)
Drug Stores & Pharmacies	-0.003	(-0.009, 0.003)	-0.014	(-0.035, 0.006)
Elementary & Secondary Schools	-0.004	(-0.017, 0.010)	0.044	(-0.016, 0.105)
Community Colleges	-0.002	(-0.131, 0.126)	0.926	(-0.106, 1.957)
Churches	0.003*	(0.000, 0.006)	-0.004	(-0.019, 0.010)

Separate regression models used for unadjusted and adjusted associations. All models include fixed effects for state and clustered standard errors

Regressions were run in Stata 16.1 using menbreg and mixed commands

*CCVI* COVID Community Vulnerability Index

*CI* Confidence Interval

^*^p < .05 **p < .01 ***p < .001

When the separate thematic sub-scores and availability of community resources were entered simultaneously, population density, socioeconomic status, minority status and language, and the number of churches were positively associated with density of both testing and vaccination sites, while healthcare system factors and high-risk environments were negatively associated with density of both testing and vaccination sites. Additionally, the score for epidemiological factors was negatively associated with density of non-private testing sites, and the number of physicians’ offices was negatively associated with density of non-private vaccination sites.

In the final set of models including the separate thematic sub-scores and availability of community resources, population density also was strongly negatively associated with both average distance to nearest testing site and distance to nearest vaccination site. Housing type and household composition score was negatively associated with average distance to nearest testing site, while healthcare system factors and the number of churches were associated with greater average distance to COVID testing. Finally, in contrast to the model for accessibility of COVID testing, when accounting for population density, healthcare system factors also were negatively associated with distance to nearest vaccination site.

## Discussion

Using a national database on recovery housing, this study documented that indicators of higher vulnerability to COVID were positively associated with recovery housing availability and the number of recovery residences in counties with at least one recovery residence. Although our findings underscore potential risks faced by individuals living in recovery housing, they also highlight effective targeting of recovery housing in areas that have higher population density, more housing risk factors and other high-risk environments related to the spread of an infectious disease such as COVID. Some research suggests that rates of COVID infection and mortality may have been lower among recovery housing residents than rates in the general population [29]. While more research is needed, similar findings from a broader sampling of recovery residences could provide additional evidence for potentially salutary effects of recovery housing as a part of a robust continuum of care contributing to the health and wellbeing of those living in the houses, as well as to those in the communities where residences are located.

In addition to examining the association between overall COVID vulnerability and the availability of recovery housing, we also examined additional components of the CCVI corresponding to key SDOH, as well as other community resources. In bivariate analyses, counties with recovery housing were found to have lower epidemiological and healthcare system factors scores but more resources, like physicians’ offices, drug stores, educational settings, and churches. Prior work examining availability of recovery housing found that residences were more likely to be in urban areas [31], and it is likely that these other supportive resources would also be more prevalent in urban areas. Indeed, in multivariate models including population density scores, many of these differences were no longer significant, except for associations between healthcare system factors scores and density of churches with both recovery housing availability and density. Future research examining the role of these factors in relation to recovery housing and health risk may be particularly useful in thinking about the role that recovery housing could play as a health resource.

While it is heartening that recovery housing can be found in more densely populated counties, greater attention should be paid to ensuring that recovery housing can also be accessed by individuals in less densely populated areas, which may have fewer recovery and healthcare services [41]. Further, the negative association between healthcare system factors scores and recovery housing signals potential relegation of recovery housing to lesser-resourced areas within urban areas, as the positive association between high-risk environment and recovery housing availability would suggest. One encouraging finding is that the indicator of racial, ethnic and immigrant minority status was positively associated with the number of recovery residences in counties that had at least one residence. Although this could be an artifact of recovery residence locations in urban areas, this contrasts with national studies of Medicaid substance use treatment facilities that demonstrate marked lack of availability in areas with higher concentrations of racial and ethnic minoritized and marginalized populations [42].

This study also examined factors associated with the COVID response, using geocoded data on counts and mean distances to COVID testing and vaccination sites in counties with recovery housing. In these areas, greater COVID vulnerability was associated with greater numbers of testing and vaccination sites, as well as shorter distances to these resources. Again, this finding highlights potentially beneficial targeting of recovery housing to areas with greatest need, but this also could be heavily influenced by the increased availability of recovery housing in densely populated areas. Indeed, in models including the CCVI’s separate thematic sub-scores and availability of community resources, population density was negatively associated with both average distance to nearest testing site and distance to nearest vaccination site, suggesting greater accessibility in more densely populated areas. Further, when accounting for population density, healthcare system factors were positively associated with distance to nearest testing site, yet again suggesting that recovery housing may be relegated to under-resourced neighborhoods within urban areas. Density of churches also was positively associated with density of testing and vaccination sites; as such, churches may be an important, albeit less-traditional, resource to consider when addressing spread of infectious diseases among communities with recovery housing.

This study represents the first examination of COVID vulnerability and recovery housing across the US, but a number of key limitations to this work should be noted. Information on recovery residences came from a database of over ten thousand residences across the country, but this information was collected in 2020 and may not represent an exact count of all residences due to underreporting and inclusion of residences that may have subsequently closed [31]. Further, although we included covariates in our models to capture other key health resources, reporting of COVID testing and vaccination sites is voluntary and may reflect an undercount of resources available within counties; because we expect that any undercounting would be non-differential with respect to county-level recovery housing, potential bias is likely negligible. Additionally, our measures of COVID vulnerability represent composites of a number of factors, and some of the themes, like the healthcare system factors score, mix county and state-level components, which present more challenges when interpreting findings from these scores. Finally, although our results accounted for population density in the CCVI measure (Theme 7), it also may be that some of the associations are due to relationships of recovery housing and COVID testing and vaccination sites with urbanicity or population size. However, it should also be noted that our findings were substantively robust to alternative model specifications accounting for a county’s status as urban, adjacent rural or non-adjacent rural, and sensitivity analyses using density measures of the other community resources suggested the models adequately accounted for county size. Future research on this topic should examine the relative contributions of individual measures of key SODH.

## Summary and conclusions

Recovery residences tend to be located in areas highly vulnerable to COVID. While this may present a higher risk for people in recovery, these communities could benefit from the support and resources provided by recovery residences, which may help offset individual-level COVID risks. Additionally, recovery housing residents may benefit from proximity to local COVID testing and vaccination resources, particularly those in more densely populated areas with higher socioeconomic vulnerability and more racial and ethnic diversity. Future research should explore the extent to which residents in recovery residences located in vulnerable areas were affected by the COVID pandemic, as well as the role of supportive community resources and the relative contributions of individual measures of key SODH in addressing the needs of individuals in recovery housing.

## Data Availability

Contents of the NSTARR database will be available through data use agreements at the completion of the NSTARR project. This study also used data sources that are publicly available. COVID-19 Community Vulnerability Indices come from https://precisionforcovid.org/ccvi; COVID Testing and Vaccination Sites data come from https://covid-19-giscorps.hub.arcgis.com/pages/contribute-covid-19-testing-sites-data; County Business Patterns (NAICS) data (Community Resources) come from the Census Bureau website: https://www.census.gov/data/developers/data-sets/cbp-nonem

## Abbreviations

CCVI: Community COVID Vulnerability Index
COVID: Coronavirus disease
FIPS: Federal Information Processing System
NAICS: North American Industry Classification System
NIAAA: National Institute on Alcohol Abuse and Alcoholism
NSTARR: National Study of Treatment and Addiction Recovery Residences
PRR: Prevalence rate ratio
SDOH: Social determinants of health
SUD: Substance use disorder
URISA: Urban and Regional Information Systems Association

## Declarations

### Competing and interests

No authors have no competing or conflicting interests to declare.
